# Exploring the function of myeloid cells in promoting metastasis in head and neck cancer

**DOI:** 10.37349/etat.2024.00208

**Published:** 2024-02-19

**Authors:** Dakota Dike Dimegwu Okwuone, Deri Morgan, Gregory N. Gan

**Affiliations:** Istituto Nazionale Tumori-IRCCS-Fondazione G. Pascale, Italy; ^1^Department of Cancer Biology, University of Kansas Medical Center, Kansas City, KS 66160, USA; ^2^Department of Radiation Oncology, University of Kansas Medical Center, Kansas City, KS 66160, USA

**Keywords:** Head and neck cancer, metastasis, immunology, tumor microenvironment, macrophages, neutrophils, dendritic cells

## Abstract

Head and neck cancer (HNC) is a challenging disease that lacks effective treatment, particularly in the cases that spread locoregionally and metastasize distantly, dramatically reducing patient survival rates. Expanding the understanding of the mechanisms of the metastatic cascade is critical for creating more effective therapeutics that improve outcomes for HNC patients. A true grasp of cancer metastasis requires the consideration of all cell types that contribute to the inflammatory HNC microenvironment as drivers of this process. More emphasis now is being placed on exploring the roles of the different immune cells in cancer control, tumorigenesis and metastasis. Myeloid cells are the most numerous immune cell types in the body, and they are actively recruited and reprogrammed by tumor cells to behave in a variety of ways. These cells are remarkably diverse in phenotype and function, and the part they play in tumor spread greatly differs based on the cell type. This review will focus on summarizing the roles of macrophages, neutrophils, myeloid derived suppressor cells (MDSCs), and dendritic cells (DCs) in driving HNC metastasis by examining the current knowledge base and offering potential new routes through which to target and treat this deadly process.

## Introduction

Head and neck cancer (HNC) afflicts nearly 900,000 new patients every year worldwide, including 66,000 in the United States, making it the 6th most common cancer globally [1]. HNC is an amalgam of cancers found in the oral cavity, nasal cavity, paranasal sinuses, pharynx, larynx and salivary glands. Over 90% of these can be characterized as head and neck squamous cell carcinoma (HNSCC) [2]. The survival rate of HNC patients differs based many factors, including where in the head and neck region the tumor arises, the type of cancer, comorbidities of the patient (i.e. alcohol abuse, smoking history) and whether or not the case can be attributed to a human papillomavirus (HPV) infection [approximately 15% of HNC patients are HPV^+^]. Nevertheless, a common fact between these tumors is that metastasis (or spread to another site), whether locoregionally within the surrounding tissue, to lymph nodes or to distant secondary sites such as the lung, drastically reduces overall survival (OS) inpatients [3]. Metastatic spread requires the completion of several sequential steps, including tumor cell migration/invasion, intravasation into lymph or blood vessels, extravasation into a premetastatic niche, and finally secondary site colonization [4]. Improving the understanding of critical factors in the metastatic cascade and devising strategies to interfere with its progression is of the utmost importance for ameliorating HNC patient survival.

Given the anatomical location where HNC develops, the continual trauma and rapid cellular proliferation that occurs with normal tissue, and the common use of cytotoxic therapies (i.e. radiation, chemotherapy) to manage this disease, it is clear that inflammation and the immune system are linked to HNC tumorigenesis, progression, and metastasis [3, 4]. The last couple of decades of research has greatly improved our understanding of the role of the immune microenvironment in cancer, and this is especially true for myeloid cells including macrophages, neutrophils, myeloid derived suppressor cells (MDSCs), and dendritic cells (DCs). These immune cells have a myriad of effects on tumors and are both cancer-promoting and cancer-suppressing. This review will focus on the characterization and recruitment of macrophages, neutrophils, MDSCs, and DCs and the role they play in facilitating HNC spread and metastasis.

## Macrophages

Macrophages have been associated with a multitude of diverse functions in cancer depending on their function and cellular markers. They are a spectrum of different macrophage phenotypes, but the 2 types that have garnered the most study are M1s and M2s. M1s, which characteristically express markers CD80^+^/CD86^+^/inducible nitric oxide synthase (iNOS^+^), behave like classic antigen-presenting cells (APCs) and are more anti-tumorigenic through direct phagocytosis and cytotoxicity, as well as via secretion of interferon-gamma (IFN-γ) and other pro-inflammatory cytokines leading to cytotoxic T lymphocyte activation [5]. In contrast, M2 macrophages are more pro-tumorigenic and are typically characterized by CD163^+^/CD206^+^/arginase 1 (Arg1^+^) markers [5, 6]. In a meta-analysis of eight independent studies by Kumar et al. [7], they showed that increased total (CD68^+^) tumor-associated macrophages (TAMs) and M2-like macrophages (CD163^+^) were associated with worse T-stage, lymph node metastasis, and vascular invasion. This observation of the quantity of total and M2-like TAMs being negatively correlated with OS has also been corroborated in another review and meta-analysis, highlighting the need for further mechanistic examination of the impact of TAMs on tumor progression [8].

The mobilization and polarization of macrophages in the tumor microenvironment is presumably controlled by the secretion of inflammation-mediating cytokines and chemokines from tumors, either from the cancer cells themselves or other cells in the surrounding stroma. These factors can directly control the recruitment and activation of immune cells in the primary cancer site as well as systemically throughout the body. Keying in on specific secreted factors and their effects particular cell populations will be invaluable to devising future therapeutic interventions. For macrophages, a plethora of cytokines/chemokines have been shown to cause their recruitment, including interleukin-1α (IL-1α), IL-1β, IL-8, IL-10, IL-34, C-C motif chemokine ligands 2 (CCL-2), macrophage colony-stimulation factor (M-CSF)/colony-stimulation factor-1 (*CSF-1*), and granulocyte-M-CSF (GM-CSF)/*CSF-2* [9, 10]. Cytokines also drive macrophage activation and macrophage polarization with IFN-γ and tumor necrosis factor-α (TNF-α) established as activators of M1 TAM differentiation and M-CSF, IL-4, IL-10, IL-13, and IL-34 inducing M2 polarization [10, 11]. IL-4 and IL-6 have been used to stimulate the differentiation of CD163^+^ M2-like TAMs from primary human monocytes, and conditioned media from these resulting TAMs increased HNC tumor cell migration, invasion, and angiogenesis *in vitro* [12].

New insights into the function of TAMs in HNC progression have brought forth an array of regulatory mechanisms. One of these mechanisms includes the induction of epithelial-to-mesenchymal transition (EMT) by TAMs. EMT involves the shift in the phenotype of tumor cells from a more epithelial morphology, characterized by high proliferation and cell-cell adhesion, to a more mesenchymal phenotype with increased motility, invasion, and stemness [13]. This process has been strongly correlated with nodal and distant metastasis in HNC [13, 14]. Conditioned media from TAMs was shown to induce shifts in the expression of EMT-related genes (down-regulation of epithelial marker E-cadherin, and upregulation of mesenchymal marker vimentin) in HNC cells while enhancing their migratory and invasive ability [15]. This aspect of macrophage function on tumor cells is not specific to the M2 phenotype as, somewhat contrary to conventional thought, M1-like TAMs (CD80^+^/CD86^+^) have also been shown to induce EMT, migration, and invasion in HNC cells by secreting IL-6 and activating signal transducer and activator of transcription 3 (STAT3) in the tumor cells [16].

In addition to causing tumor cell morphological changes, TAMs have been implicated in the regulation of vessel architecture in the tumor stroma. High levels of peri- and intratumoral macrophages are associated with lymph node metastasis and vessel invasion [7, 8]. Yamagata et al. [17] showed that CD163^+^ (M2-like) macrophages were correlated with increased lymphatic vessel density in HNC patient samples, suggesting that they promote lymphangiogenesis, likely through the secretion of vascular endothelial growth factor-C (VEGF-C), a major factor for the growth of lymphatic vasculature. Lymphangiogenesis is a significant factor in metastatic spread to lymph nodes [18], making this another route through which TAMs can impact HNC progression towards distant growth.

## Neutrophils

Recent seminal studies have exposed the clinical and mechanistic relevance of neutrophils in cancer progression. In a manner similar to macrophages, tumor-associated neutrophils (TANs) can be subdivided into antitumor N1s and protumor N2s subtypes. These subtypes can be differentiated from general neutrophils [classically expressing CD66b^+^, CD11b^+^ for humans and CD11b^+^, lymphocyte antigen 6 complex, locus G (Ly6G^+^) for mice] by the expression of proinflammatory signals CCL-3, C-X-C motif ligand (CXCL) 9/10, TNF-α, IL-12, GM-CSF and cytotoxic iNOS for N1s, whereas N2s secrete proangiogenic protein VEGF, extracellular matrix remodeling enzyme matrix metalloproteinase 9 (MMP9), immunosuppressive protein Arg1, and other immunosuppressive cytokines [19]. Numerous retrospective studies have demonstrated that both high absolute neutrophil counts and high circulating neutrophil-to-lymphocyte ratios are poor prognostic features and serve as markers for treatment resistance, tumor recurrence, and locoregional and distant disease progression in HNC [20, 21]. High levels of TANs in various forms of HNC have also been linked with decreased overall and disease specific survival and lymph node metastatic risk inpatients [22, 23]. These observations are also seen in various other cancer types, including several gastroenterological malignancies (i.e. esophageal, colorectal, and pancreatic), breast cancer, melanoma, and bladder cancer [24–26], showing that neutrophils play an important part in overall cancer progression.

Neutrophil production and recruitment into circulation and to a tumor is regulated by alarge host of inflammatory signals. These signals include GM-CSF, granulocyte CSF (G-CSF/*CSF-3*), chemokines CXCL1, CXCL2, CXCL8, and cytokines IL-6 and IL-8, which drive granulopoiesis, the accumulation of circulating neutrophils, and subsequent honing of the cells to specific locations in the body [19, 27]. Transforming growth factor-β (TGF-β) is a powerful regulator of neutrophil polarization. Fridlender et al. [28] showed that blocking TGF-β signaling *in vivo* caused greater neutrophil infiltration into tumors, with these cells being more cytotoxic, more hyper-segmentation, and having greater expression of pro-inflammatory cytokines (i.e. TNF-α and IFN-γ), all of which are the defining characteristics of N1 neutrophils. New studies have described the role of IFN-β in promoting N1 polarization [29]. In contrast, G-CSF has been shown to preferentially drive the production of T cell-suppressing, pro-tumor CD11^+^Ly6G^+^ neutrophils in breast cancer [30].

TANs within the adjacent tumor microenvironment can have multiple pro-tumorigenic functions. Coculturing HNC cell lines with (activated) neutrophils led to increased tumor cell invasion by inducing invadopodia formation [31]. Another study showed that co-culture of neutrophils with HNC cell lines lead to increased cancer cell proliferation, migration, invasion, and *EMT* gene expression (including EMT-mediating transcription factors snail and slug) through a chemerin/janus kinase 2 (JAK2)/STAT3 cascade, potentially leading to cancer progression [32]. In addition to the regulation of tumor motility, neutrophils have been implicated in inducing angiogenesis in multiple tumor types [33, 34], which is another route through which these myeloid cells can promote tumor growth and spread [35]. However, it has not yet been established that they perform this function specifically in HNC.

Direct association with tumor cells at the primary site is not the only way that neutrophils can impact cancer metastasis. Circulating neutrophils have been reported to produce neutrophil extracellular traps (NETs), which are webs of sticky DNA and proteases (e.g., myeloperoxidase) that neutrophils can extrude and use to entrap and kill different pathogens [36]. In various forms of cancer, NETs have been associated with the development of pre-metastatic niches in secondary organs, including the lungs and the liver [37, 38]. The release of proteases during NET formation activate many pro-tumor inflammatory signals while the viscous, NET-like DNA attract, ensnare and support circulating tumor cells, enhancing the retention of tumor cells in the organ and thus creating a niche for tumor cell colonization. In HNC specifically, NET formation both at the primary tumor site and inpatients’ blood has been demonstrated to be a poor prognostic factor [39, 40], but the mechanistic understanding of the molecular process and its contribution to adverse HNC patient outcomes and metastases is lacking, necessitating additional study.

## MDSCs

MDSCs come from myeloid progenitors that have not fully differentiated and are aptly named for their capacity to suppress the activity of cytotoxic T cells. As this results in the promotion of tumor growth, there is a growing interest in understanding the function of MDSCs in cancer progression [41]. These cells can be split into two cell types: granulocytic/polymorphonuclear MDSCs (PMN-MDSCs) and monocytic MDSCs (M-MDSC). PMN-MDSCs are more similar to neutrophils due to their shared lineages, with markers CD11b^+^, CD14^−^, CD15^+^/CD66b^+^ in humans and CD11b^+^, Ly6C^low^, Ly6G^+^ in mice. M-MDSCs share their precursors with monocytes like macrophages, expression markers CD11b^+^, CD14^−^, CD15^–^/CD66b^–^ in humans and CD11b^+^, Ly6C^high^, Ly6G^–^ in mice [41]. The exact differences between the functions of these two subgroups are still being elucidated but they both serve to suppress both the innate and the adaptive immune system.

MDSCs, which are fundamentally immature monocytes/neutrophils, share several of the same cytokine signaling pathways as their counterparts. VEGF, IL-1β, IL-6, IL-17, GM-CSF, and TNF-α are some of the factors that can induce MDSC production and activity [9]. CCL-2, through its receptor C-C chemokine receptor 2 (CCR2), is a significant signal in the recruitment and immunosuppressive activity of MDSCs [42], preferentially recruiting the M-MDSC subtypes [43]. The mobilization PMN-MDSCs occurs through CXCL1, CXCL2, and CXCL5 signaling via their shared receptor C-X-C motif receptor 2 (CXCR2) [44].

Aside from their T cell-inhibiting abilities, MDSCs can directly influence the metastatic process in HNC in several ways. At the primary site, CD33^+^ (an older general MDSC marker) cell infiltration was found to be associated with poor prognosis inpatient pathology in samples with oral squamous cell carcinoma (OSCC) [45]. The tumor-infiltrated CD33^+^ MDSCs increased tumor cell migration, invasion, EMT, and vasculogenic mimicry in *in vitro* co-culture models [45]. These myeloid cells have also been implicated in premetastatic niche development. In the 2012 study by Sceneay et al. [46], it was observed that tumor hypoxia-induced systemic recruitment of CD11b^+^/Ly6C^med^/Ly6G^+^ myeloid cells, likely PMN-MDSCs, established a premetastatic niche in the lungs of mice. This niche formation occurred when the mice were treated intraperitoneally with conditioned media from primary mammary or melanoma tumor cells in hypoxic conditions (HCM), as opposed to normoxic conditions (NCM). Within these lungs, the MDSCs suppressed the differentiation and cytotoxicity of CD3^−^/natural killer (NK)1.1^+^ cells, rendering the lungs more conducive to metastasis. Intriguingly, when mice treated with mammary tumor cell HCM were intravenously injected with either mammary or melanoma tumor cells, both cell types exhibited increased lung metastatic burden compared to NCM-treated mice. This observation suggests that the premetastatic lung changes induced by HCM treatment are applicable across different cancer types. Several other studies have supported the hypothesis of metastatic priming of the lungs by monocytic and granulocytic MDSCs, albeit by multiple mechanisms [47, 48]. Not many studies have been done to show this same phenomenon in the setting of HNC, but there is plenty of justification for further exploration of MDSCs in HNC metastasis (Table 1).

**Table 1:** Polarization and identification of macrophage, neutrophil, and MDSC subtypes

**Cell type**	**Subtypes**	**Polarizing cytokines**	**Differentiating markers**
Macrophages	M1	IFN-γ, TNF-α	CD80^+^, CD86^+^, iNOS^+^
	M2	M-CSF, IL-4, IL-6, IL-10, IL-13, IL-34	CD163^+^, CD206^+^, Arg1^+^
Neutrophils	N1	IFN-β	Expression of: CXCL9, CXCL10, TNF-α, IL-12, GM-CSF, iNOS
	N2	TGF-β, G-CSF	Expression of: VEGF, MMP9, Arg1
MDSCs	PMN-MDSCs	CXCL1, CXCL2, CXCL5	Human: CD11b^+^, CD14^–^, CD15^+^/CD66b^+^ Mouse: CD11b, Ly6C^low^, Ly6G^+^
	M-MDSCs	CCL-2	Human: CD11b^+^, CD14^–^, CD15^–^/CD66b^–^ Mouse: CD11b^+^, Ly6C^high^, Ly6G^–^

## DCs

The canonical role of DCs is antigen processing and presentation for effector T cells, initiating T cell-mediated tumor targeting and activation. However, recent cancer-focused studies suggest they have a more nuanced role than was previously assumed. The overall impact of tumor infiltration by DCs on patient survival and disease progression remains inconclusive, in part, due to the diverse subtypes of DCs [49]. DCs can also be subdivided into different groups: conventional DCs (cDCs) types 1 and 2, plasmocytoid DCs (pDCs), and monocyte-derived DCs (moDCs) [45] cDCs types 1 & 2 behave like classic antigen-presenting, T cell-activating cells, with the main difference between them being that type 1 cells express major histocompatibility complex class I (MHC I), presenting intracellular antigens to CD8^+^ cells, and type 2 presenting extracellular pathogens to CD4^+^ cells through MHC II molecules [38] and they can be both circulating and resident in different tissues. pDCs’ responsibility centers around sensing intracellular viral or self-antigens and eliciting an IFN response; however, they have the capacity to become tolerogenic, meaning they promote immune tolerance through being immunosuppressive [49, 50]. moDCs, also referred to as inflammatory DCs, have been shown to be essential for cytotoxic T-cell activation and helper T-cell response [51].

DC expansion, migration, and maturation are very well-regulated, as the cells must travel to various tissue sites, gather antigens, take and present them in lymphoid tissues, and finally activate T cells and drive an immune response. DCs will transiently express certain chemokine receptors depending on their function and destination [52]. The DC receptor CCR7 plays the crucial role of controlling trafficking to lymph nodes for subsequent contact with lymphocytes through ligands CCL-19, CCL-20, and CCL-21 [52]. For DC development, the cytokine Fms-like tyrosine kinase 3 ligand (Flt3L) is a major factor, as it is needed for the growth of functional cDCs and pDCs, but not moDCs [53]. GM-CSF is another important cytokine for this cell type, regulating tissue-resident cDC development and antigen presentation to CD8^+^ T cells [54]. Identification of signals regulating pDC activation is a work in progress. A major responsibility for pDCs is the secretion of IFNs, specifically, IFN-α, making this a good proxy for their activity. Activated pDCs cultured with media from HNC cells or individually treated with IL-10 showed significantly inhibited IFN-α production [55]. The use of an antibody against the IL-10 receptor on the pDCs treated with the media from cancer cells did not significantly block the IFN-α suppression, so there must be other factors at play that influence the phenotype of these cells.

**Figure 1:**
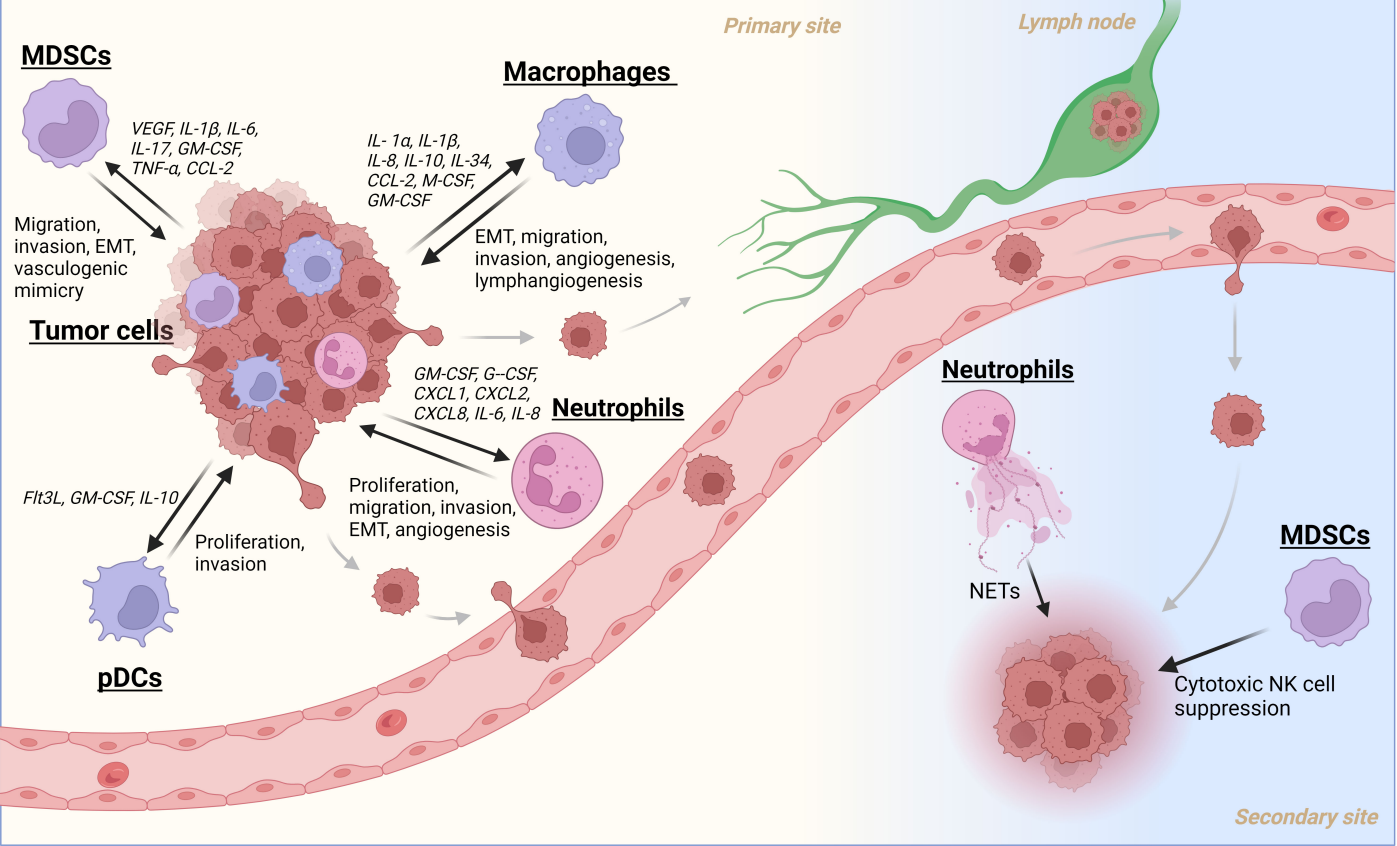
A summary of myeloid cell recruitment/activation and their subsequent contributions to the metastatic cascade in HNC. Created with BioRender.com

Current literature implies that pDCs play a supporting role in cancer metastasis. O’Donnell et al. [50] used immunohistochemistry (IHC) to quantify CD123^+^ pDCs and found elevated levels of them inpatient tumors were associated with worse OS. No change in survival was seen for other groups of DCs in their study. Han et al. [56] also found that tumor infiltration of pDCs was a poor prognostic marker and was significantly linked to lymph node metastasis. Later studies by the same laboratory showed that pDCs can enhance tumor cell proliferation and invasion *in vitro* through TNF-α secretion activating nuclear factor-kappaB (NF-κB) and CXCR4 chemokine receptor in tumor cells [57]. More investigation must be done to fully ascertain the ways that these cells promote HNC progression, but these studies suggest pDCs maybe an important immune cell type to study and target for metastatic control (Figure 1).

## Therapeutic targeting of myeloid cells in HNC

Treatment for disseminated HNC predominantly involves systemic therapies such as chemotherapy (i.e. cisplatin), targeted therapy [i.e. epidermal growth factor receptor (EGFR)-targeted monoclonal antibody (mAb) cetuximab], or more recently, immunotherapy [anti-programmed death protein-1 (PD-1) mAbs pembrolizumab and nivolumab], since surgery and radiation therapy may have limited effectiveness in advanced stages [58]. Despite these treatment options, there has been a lack of significant improvement in HNC survival rates over the last several decades [58]. Because of this, novel drug development is necessary, and targeted therapies directed at myeloid cells show promise in the treatment of HNC metastasis and recurrence. Pembrolizumab and nivolumab are mAbs specific for the immune checkpoint receptor PD-1. When present on T cells and stimulated by its ligand, programmed death ligand-1 (PD-L1), PD-1 leads to T cell exhaustion and tolerogenicity, resulting in pro-tumorigenic immunosuppression. These drugs inhibit this receptor-ligand interaction, rescuing anti-tumor T cell activity and ideally immune-mediated cancer killing. PD-L1 is present on the surface of both cancer cells and other stromal cells, including myeloid cells [59, 60]. Recent studies have also shown that PD-1 is expressed on progenitor myeloid cells, and *in vivo* depletion of this receptor specifically in myeloid cell results in defective immunosuppressive MDSC development and improved anti-tumor T cell activity in colon cancer models [61]. In the same vein, Klement et al. [62] found that stimulation of myeloid PD-1, not T cell PD-1, by tumor PD-L1 lead to suppressed cytotoxic lymphocyte recruitment to lung metastases in triple negative breast cancer, colon cancer, and melanoma models. Several clinical trials show promising results for the use of nivolumab and pembrolizumab in the setting of recurrent/metastatic (R/M) HNSCC [63, 64], and their direct interaction with myeloid cells maybe an underlying mechanism for their efficacy and a route for improvement.

Efforts are underway to develop novel methods of treating HNC through the modulation of myeloid cells. Moreira et al. [65] investigated STAT3 inhibition combined with toll-like receptor 9 (TLR9) receptor activation using cytidine phosphate guanosine (CpG)-STAT3 antisense oligonucleotide (ASO). The TLR9 receptor, commonly expressed on APCs, including monocytes/macrophages and DCs, responds to unmethylated CpG oligonucleotides (OGN) predominantly found in microbial DNA [66]. STAT3-regulated transcriptional activity in myeloid cells has been linked to immunosuppressive phenotypes in HNC patients [67], so this group decided to utilize a drug construct that combined the TLR9 ligand CpG and STAT3 ASO for myeloid cell-targeted inhibition of STAT3 translation/expression. Intratumoral injection of CpG-STAT3 ASO improved tumor control in both treated and distant tumors in a dual flank *in vivo* HNC model, and treatment induced tumor regression at both sites when combined with radiation [65]. This treatment approach effectively shifted macrophage polarization from tolerogenic to immunogenic, concomitant with the maturation of CD11^+^ DCs, allowing the generation of an anti-tumor immune response. Another approach involves reprogramming MDSCs using tadalafil, a phosphodiesterase-5 (PDE-5) inhibitor, which downregulates the expression of T cell-suppression factors Arg1 and iNOS [68]. When tadalafil was combined with nivolumab, it resulted in enhanced immune cell signatures associated with response to the immune checkpoint inhibitor [69]. Clinical trials combining tadalafil and pembrolizumab for treating R/M HNSCC are underway (NCT03993353), exemplifying the evolving landscape of myeloid-targeted therapies.

## Conclusions

Cancer metastasis is a known enemy hiding in plain sight; we understand that this process is responsible for causing the vast majority of cancer patient deaths, and yet, despite the best efforts of the scientific community to target metastatic disease, patients with metastatic disease are usually seen as incurable. Expanding the current understanding of cancer progression is paramount, and this means not only studying the tumor cells themselves, but the cells they interact with, including myeloid cells. From this review, it is apparent various types of myeloid cells play key roles in promoting the spread and dissemination of HNC. The emphasis in the field is shifting towards directing cancer treatments to influence immune cells, alongside cancerous cells. However, there is still a considerable gap in our understanding of these processes and the most effective strategies for targeting them. While there is still much to be done to prevent and treat tumor metastasis in head and neck patients, uncovering the contribution of macrophages, neutrophils, MDSCs, and DCs to cancer spread brings the field closer to making advanced-stage HNC a more treatable condition.

## Abbreviations

Arg1: arginase 1
ASO: antisense oligonucleotide
CCL-2: C-C motif chemokine ligands 2
cDCs: conventional dendritic cells
CpG: cytidine phosphate guanosine
CSF-1: colony-stimulation factor-1
CXCL: C-X-C motif ligand
DCs: dendritic cells
EMT: epithelial-to-mesenchymal transition
G-CSF: granulocyte colony-stimulation factor
GM-CSF: granulocyte macrophages colony-stimulation factor
HCM: hypoxic conditions
HNC: head and neck cancer
HNSCC: head and neck squamous cell carcinoma
IFN-γ: interferon-gamma
IL-1α: interleukin-1α
iNOS: inducible nitric oxide synthase
Ly6G^+^: lymphocyte antigen 6 complex, locus G
mAb: monoclonal antibody
M-CSF: macrophage colony-stimulation factor
MDSCs: myeloid derived suppressor cells
M-MDSC: monocytic myeloid derived suppressor cells
moDCs: monocyte-derived dendritic cells
NETs: neutrophil extracellular traps
OS: overall survival
PD-1: programmed death protein-1
pDCs: plasmocytoid dendritic cells
PD-L1: programmed death ligand-1
PMN-MDSCs: polymorphonuclear myeloid derived suppressor cells
STAT3: signal transducer and activator of transcription 3
TAMs: tumor-associated macrophages
TANs: tumor-associated neutrophils
TGF-β: transforming growth factor-β
TLR9: toll-like receptor 9
TNF-α: tumor necrosis factor-α
VEGF: vascular endothelial growth factor

## Declarations

### Copyright

© The Author(s) 2024.
